# Distinct Patterns of IgG and IgA against Food and Microbial Antigens in Serum and Feces of Patients with Inflammatory Bowel Diseases

**DOI:** 10.1371/journal.pone.0106750

**Published:** 2014-09-12

**Authors:** Lisa Frehn, Anke Jansen, Eveline Bennek, Ana D. Mandic, Ilknur Temizel, Stefanie Tischendorf, Julien Verdier, Frank Tacke, Konrad Streetz, Christian Trautwein, Gernot Sellge

**Affiliations:** Department of Internal Medicine III, University Hospital Aachen, RWTH University, Aachen, Germany; University of South Carolina School of Medicine, United States of America

## Abstract

**Background:**

Inflammatory bowel disease (IBD) is associated with a defective intestinal barrier and enhanced adaptive immune responses against commensal microbiota. Immune responses against food antigens in IBD patients remain poorly defined.

**Methods:**

IgG and IgA specific for food and microfloral antigens (wheat and milk extracts; purified ovalbumin; *Escherichia coli* and *Bacteroides fragilis* lysates; mannan from *Saccharomyces cerevisiae*) were analyzed by ELISA in the serum and feces of patients with Crohn's disease (CD; n = 52 for serum and n = 20 for feces), ulcerative colitis (UC; n = 29; n = 17), acute gastroenteritis/colitis (AGE; n = 12; n = 9) as well as non-inflammatory controls (n = 61; n = 39).

**Results:**

Serum anti-*Saccharomyces cerevisiae* antibodies (ASCA) and anti-*B. fragilis* IgG and IgA levels were increased in CD patients whereas antibody (Ab) levels against *E. coli* and food antigens were not significantly different within the patient groups and controls. Subgroup analysis revealed that CD patients with severe diseases defined by stricturing and penetrating lesions have slightly higher anti-food and anti-microbial IgA levels whereas CD and UC patients with arthropathy have decreased anti-food IgG levels. Treatment with anti-TNF-α Abs in CD patients was associated with significantly decreased ASCA IgG and IgA and anti-*E. coli* IgG. In the feces specific IgG levels against all antigens were higher in CD and AGE patients while specific IgA levels were higher in non-IBD patients. Anti-food IgG and IgA levels did not correlate with food intolerance.

**Summary:**

In contrast to anti-microbial Abs, we found only minor changes in serum anti-food Ab levels in specific subgroups of IBD patients. Fecal Ab levels towards microbial and food antigens show distinct patterns in controls, CD and UC patients.

## Introduction

Inflammatory bowel diseases (IBD) include a range of chronic, immune-mediated inflammatory disorders of the gastrointestinal system with fluctuating activity, most frequently represented by Crohn's disease (CD) or ulcerative colitis (UC). IBD has a multifactorial etiology with hereditary and environmental triggers and it has been associated with changes of the intestinal microflora, defects in the gastrointestinal barrier with increased transport of luminal contents into the tissue and a loss of immune tolerance [1], [2]. Consequently, specific adaptive immune responses towards luminal antigens, in particular antigens of the commensal microflora, are altered in IBD patients. Specific IgG and IgA directed against a specific oligomannose epitope present on the cell wall of the yeast *Saccharomyces cerevisiae* are strongly increased in CD patients [3], [4]. Anti-*S. cerevisiae* antibodies (ASCA) have been established as serological markers aiding in diagnosis of CD [5] and their titers correlate with the presence of ileal disease, fibrostenotic and penetrating lesions, and risk for surgery [6]. Apart from ASCA, higher titers of circulating antibodies (Abs) directed against multiple other microfloral antigens have been found in IBD and in particular in CD patients. Those antigens are for example *E. coli* outer-membrane porin C (anti-OmpC), the *Pseudomonas fluorescens*-related protein (anti-I2), a protein found in the flagella of bacteria, the CBir1 flagellin, and several other glycan epitopes or bacterial flagella as well as less well defined antigen-mixtures such as cell lysates or membrane-associated antigens of different *Bacteroides spp.*, *Klebsiella pneumoniae*, *Enterococcus faecalis* and *Candida albicans* [7]–[11].

Apart from microbial antigens, alimentary antigens are present in high concentrations in the intestinal lumen. Considering the fact that a defective intestinal barrier and a loss of immune tolerance are pathogenic factors in IBD, it is possible that also food-specific Ab levels are increased in IBD patients and may participate in immune-mediated food intolerance in IBD patients. Classical food-allergy is mediated by specific IgE antibodies and some reports suggested that IgE-mediated food allergies are more frequent in IBD patients [12]–[14]. However, specific IgE antibodies were not analyzed in this study. Furthermore, food-specific IgG and IgA antibodies are frequently found in patients as well as healthy individuals and their contribution to food intolerance remains a matter of debate [15], [16]. The results of the few studies analyzing food-specific IgG and IgA levels in the serum of IBD patients and controls are not fully consistent. Lerner et al. described increased IgG levels specific for cow's milk proteins in pediatric CD patients compared to UC patients and controls [17]. Bentz et al. used a multiplex ELISA method to measure 271 specific anti-food IgG and found multiple increased food-specific IgG levels in adult CD patients compared to controls [15]. In turn, Lindberg et al. and Suzuki et al. did not detect elevated levels of IgG and IgA directed against wheat, milk, or egg proteins in adult CD patients. However, Lindberg et al. found enhanced anti-gliadin IgA titers in adult UC patients and Suzuki et al. measured increased levels of IgG specific for porcine pancreatic amylase, a potential protease-resistant food derivative, in adult CD patients [18], [19].

As the majority of these smaller studies indicated rather elevated levels of distinct IgG and IgA in IBD patients, we hypothesized that chronic inflammation in the intestine is associated with the loss of immunological tolerance towards luminal antigens leading to pronounced specific Ab production. We therefore aimed at comprehensively investigating local or systemic levels of anti-microbial and anti-food Abs in IBD patients. For this purpose, we analysed in parallel serum and fecal Abs specific for dietary and microbial antigens in a cohort of IBD patients and controls. Our results reveal that on the systemic level only Abs directed against some microbial antigens are elevated in CD but not UC patients, whereas anti-food Abs showed no general alterations in IBD patients and only subtle changes in certain patient subgroups. In contrast, fecal Ab levels towards different luminal antigens were more uniformly regulated and showed distinct patterns in controls, CD and UC patients.

## Methods

### Patients

Serum and stool samples were obtained from well characterized IBD and control patients from the gastroenterology department at the University Hospital Aachen. Characteristics of the patients are depicted in Table S1 and S2. In addition to the IBD patients, three control cohorts were investigated: (a) healthy volunteers, (b) medical patients without infectious or non-infectious bowel disease, (c) medical patients with acute gastroenteritis (AGE). Pooled groups (a) and (b) served as non-inflammatory controls (Con). Duration of symptoms in the AGE group was < 4 weeks. Clinical disease activity of IBD patients was measured by the Harvey Bradshaw Index (HBI) for CD and Colitis Activity Index (CAI) for UC [20], [21]. Disease phenotypes were classified according to the Montreal classification [22] (Table S2).

### Ethical considerations

Patients were included into the study upon providing written informed consent. The study protocol was approved by the local ethics committee and conducted in accordance with the ethical standards laid down in the Declaration of Helsinki (ethics committee of the University Hospital Aachen, RWTH-University, Aachen, Germany, reference number EK 049/12).

### Antigen preparation

Albumin from chicken egg white (ovalbumin; grade II), non-fat dried milk powder, and purified mannan from *S. cerevisiae* were purchased (Sigma). Antigens were diluted in carbonate buffer pH 9.6. Commercially available wheat flour was mixed with sodium acetate buffer (sodium acetate 6 mM; acetic acid 88 mM; pH 3.8) according to a published protocol [23]. All antigens were vigorously mixed for 1 h. *E. coli* K12 DH5α and *B. fragilis* ATCC 25285 were grown over night in LB or thioglycolate medium under aerobic or anaerobic culture conditions, respectively. Cultures were washed by centrifugation (10.000 g, 5 min) three times in carbonate buffer to remove medium proteins. Glass beads with 0.3 µm diameter (Sigma) were added and tubes were vigorously shaken at 2.850 rpm for 15 min on a disrupter (Disruptor Genie, Scientific Industries, Inc.) in order to break bacterial cell walls. All antigen mixtures (except for mannan) were centrifuged for 20 min at 27.000 g to remove bacterial debris and larger molecular complexes. Supernatants were passed through a 0.2 µm filter. Protein concentrations were measured using the Bradford method. Protein yield of bacterial lysates were about 10% of the dry weight of total bacteria indicating sufficient bacterial lysis.

### Preparation of fecal samples

Fecal samples were diluted 1∶5 (w/w) with fecal dilution buffer (90 ml PBS, 10 ml 0.5 M EDTA pH 8, 10 mg soy bean trypsin inhibitor [Sigma]; 666 µl 100 mM PMSF [Sigma; dissolved in EtOH]). Samples were vigorously mixed and centrifuged at 10.000 g for 5 min. Supernatants were obtained and filtered through a 0.2 µm filter.

### ELISA

Microtitre plates (96 wells, Maxisorb, Nunc) were coated overnight at 4°C with 50 µl of antigens in carbonate buffer pH 9.6 The antigen concentrations were 100 µg/ml for mannan, 10 µg/ml for ovalbumin, wheat, milk, as well as *E. coli* lysate, and 1 µg/ml for *B. fragilis* lysate. For the measurement of background binding, plates without coated antigens were used. All following steps were performed at room temperature unless stated differently. Reagents, sera and fecal lysates were diluted in PBS/bovine serum albumin (BSA) 1%. Between all following steps, microtitre plates were washed four times with 200 µl of PBS/BSA 0.1%/TWEEN 0.05% using an ELISA washer (Nunc). Plates were blocked with 200 µl PBS/BSA 5% for 1 h. In a next step, plates coated with bacterial lysates were incubated with 50 µl avidin/biotin blocking reagent (Vector laboratories) for 30 min to prevent non-specific streptavidin binding. Subsequently, different dilutions of 50 µl serum or fecal homogenates were added in duplicates (serum IgG: 1∶400, 1∶1.600, and 1∶6.400; serum IgA: 1∶100 and 1∶800; stool IgG and IgA: 1∶35 final dilution [1∶5 pre-dilution as described above and 1∶7 further dilution]; higher dilutions for sera or feces if required). A 2-fold serial dilution curve of a serum with known high reactivity for the respective antigen served as standard. Serum and standard were incubated overnight at 4°C. The following incubation steps were: 100 µl of H_2_O_2_ for 10 min for blocking of endogenous peroxidases (only in plates coated with bacterial lysates), 50 µl anti-human IgG (100 ng/ml; BD Pharmingen; Clone G18-145) or anti-human IgA (500 ng/ml; BD Pharmingen; Clone G20-359) for 2 h; 50 µl streptavidin-horseradish peroxidase solution (R&D Systems) for 40 min; 100 µl TMB (3,3′ – 5,5′-Tetramethylbenzidin; Sigma) substrate solution (1 mg/ml in 0.05 M phosphate-citrate buffer, pH 5.0) for 10–15 min. The reaction was stopped by adding 50 µl 2N H_2_SO_4_. Optical density was read photometrically at 450 nm.

Total fecal IgG and IgA were measured in 1∶50 and 1∶1000 diluted fecal homogenates with commercially available ELISA kits according to manufacturer's instructions (eBioscience).

### Analyses and statistics

Specific IgG and IgA levels in serum and feces are expressed as arbitrary units (AU). The AU assigned to the standard serum were defined by the dilution at which the OD was at least two times higher than the background OD of the buffer control (dilution  =  AU). The three times standard deviation (SD) of at least 10 independent background measurements was always below two times of the background OD level. The two times background OD was also set as the detection limit. For analysis, background OD levels of the respective samples or standards measured on plates without coating of specific antigens (only blocking with BSA) were substracted from the ODs of the antigen-specific measurement. Samples were analysed at different dilutions (see above). The OD of the highest dilution above the detection limit was used for calculation of the AU. The calculated AU was corrected by the dilution factor. If all measurements were above the upper limit of detection, measurements of higher dilutions were performed.

Serum values are represented as median. Boxes and whiskers represent 25/75 percentiles and 10/90 percentiles, respectively. Statistical significance was analysed by the Mann Whitney U test for the comparison of two groups and by the Kruskal-Wallis test followed by a Dunn's post hoc test for the comparison of multiple groups. Correlations were analyzed by the Spearman correlation test.

Since specific antibodies were only detectable in less than 50% of the samples, the results are represented as percentage of measurements above the detection limit followed by a Chi-square test. In order to have a quantitative analysis, results are additionally represented as geometric means ± 95% confidential interval followed by statistical significance analysis using the Kruskal-Wallis test and Dunn's post hoc test.

Statistical analyses were performed using SPSS and GraphPad Prism.

## Results

### Anti-food and anti-microbial IgG and IgA in serum

**Figure 1:**
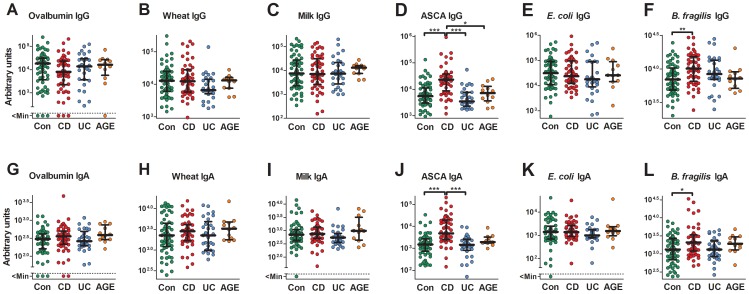
Serum IgG and IgA levels specific for food and microbial antigens in IBD patients and controls. Serum IgG (A–F) and IgA (G–L) specific for ovalbumin (A/G), wheat (B/H), milk (C/I), mannan from *S. cerevisiae*  =  ASCA (D/J), and lysates from *E. coli* K12 (E/K) and *B. fragilis* ATCC 25285 (F/L) were quantified by ELISA in control patients/healthy controls (Con; n = 61) and patients suffering from CD (n = 52), UC (n = 29) and acute gastroenteritis/colitis (AGE; n = 12). Each dot represents one patient. Medians with interquartile ranges are indicated. P values (*<0.05; **<0.01; ***<0.001) were determined by Kruskal-Wallis test followed by a Dunn's post hoc test.

IgG and IgA Abs specific for ovalbumin, wheat, milk, *S. cerevisiae*-derived mannan, *E. coli* lysate, and *B. fragilis* lysate were quantified by ELISA in the serum of patients suffering from CD, UC, patients with non-IBD acute gastroenteritis/colitis (AGE) as well as age-matched controls without inflammation in the gastrointestinal tract. IgG and IgA Abs specific for all six antigens were detectable in the majority of patients and controls, although there was a high variation in the amount of detectable Abs (Figure 1). ASCA and anti-*B. fragilis* IgG and IgA levels were significantly increased in CD patients while specific IgG and IgA for ovalbumin, wheat, milk, and *E. coli* showed no significant differences within the patient groups and controls (Figure 1).

There was a strong correlation (r_s_ = 0.48–0.73) between levels of IgG directed against different food antigens within single individuals while the correlation between different anti-microbial IgG or between anti-food and anti-microbial IgG was generally lower (Figure F1A). For IgA, the correlation between different Ab levels within single individuals was in most cases between 0.2 and 0.6 regardless of their specificity (Figure S1B). The correlation between IgG and IgA levels for the same antigen was low and not significant for ovalbumin and wheat, whereas generally stronger correlation rates could be found for milk, ASCA, *E. coli*, and *B. fragilis* (Figure S1C).

### Anti-food and anti-microbial serum IgG and IgA in patient subgroups

Anti-food IgG, but not anti-microbial IgG, correlated negatively with the age of controls and patients (r_s_ = −0.15–−0.5), while most specific IgA tended to correlate positively with the age of individuals (r_s_ = 0.34–0.61) (Figure S2).

**Figure 2:**
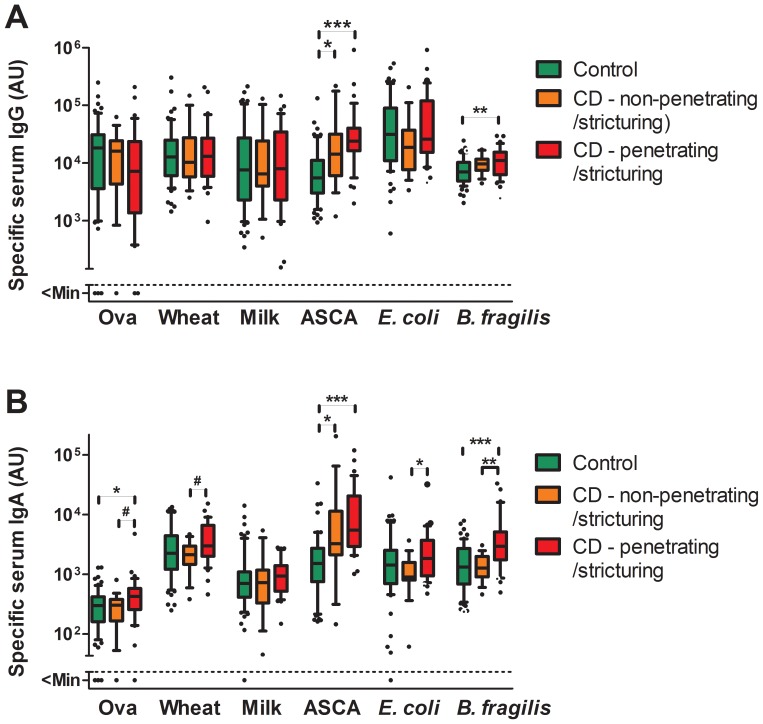
Anti-food and anti-microbial serum IgG and IgA levels in CD patients with or without stricturing/penetrating disease and controls. Specific serum IgG (A) and IgA (B) were quantified by ELISA in control patients/healthy controls (n = 61) and CD patients without (n = 17) and with (n = 34) stricturing and/or penetrating complications. Boxes indicate median and 25/75 percentiles and whiskers indicate 10/90 percentiles. P values were determined by Kruskal-Wallis test followed by a Dunn's post hoc test (*<0.05; **<0.01; ***<0.001) or by Mann Whitney U test (# <0.05). Mann Whitney U test was only applied between CD subgroups and results are only shown if the Dunn's post hoc test did not show any significance.

**Figure 3:**
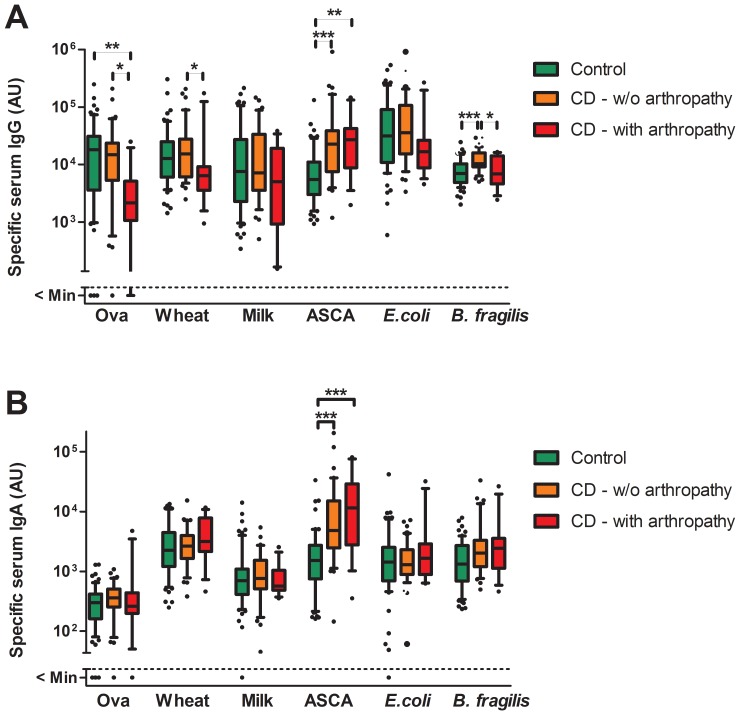
Anti-food and anti-microbial serum IgG and IgA levels in CD patients with or without arthropathy and controls. Specific serum IgG (A) and IgA (B) were quantified by ELISA in control patients/healthy controls (n = 61) and CD patients without (n = 39) and with (n = 12) current arthropathy. Boxes indicate median and 25/75 percentiles and whiskers indicate 10/90 percentiles. P values were determined by Kruskal-Wallis test followed by a Dunn's post hoc test (*<0.05; **<0.01; ***<0.001).

CD patients with stricturing and/or penetrating (fistulas, abscesses) disease displayed significantly higher amounts of anti-*E. coli* and anti-*B. fragilis* IgA than non-stricturing/penetrating CD patients, and higher amounts of anti-*B. fragilis* IgG and IgA than controls (Figure2). Interestingly, stricturing/penetrating CD patients also had significantly higher levels of anti-ovalbumin and anti-wheat IgA than non-stricturing/penetrating CD patients or controls while, conversely, arthropathy was associated with significantly decreased levels of anti-ovalbumin and anti-wheat IgG (Figure 3). Significantly decreased levels of anti-ovalbumin and anti-milk IgG were also observed in UC patients with arthropathy (Figure S3).

**Figure 4:**
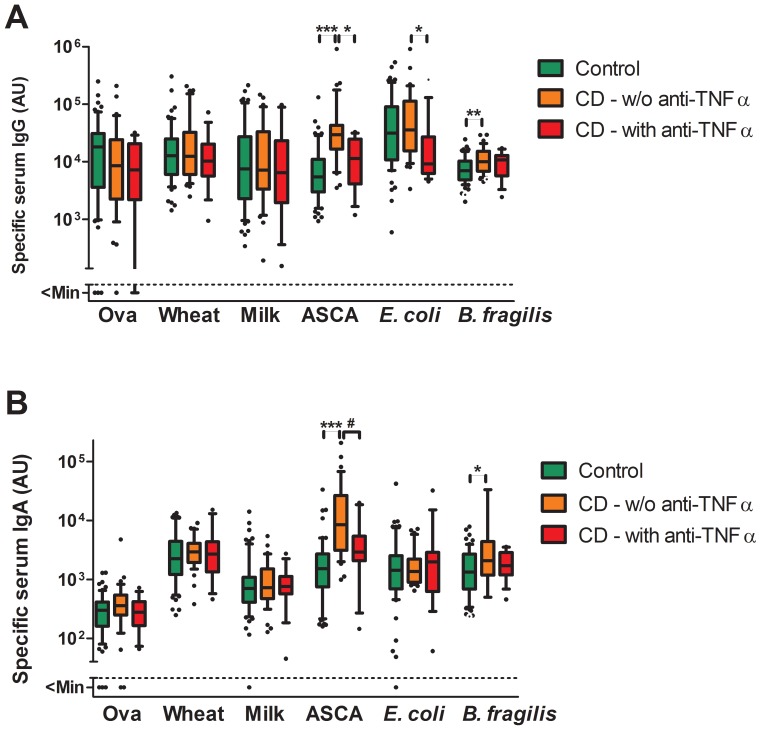
Anti-food and anti-microbial serum IgG and IgA levels in CD patients with or without anti-TNFα treatment and controls. Specific serum IgG (A) and IgA (B) were quantified by ELISA in control patients/healthy controls (n = 61) as well as in CD patients without (n = 17) and with (n = 34) current anti-TNFα treatment. Boxes indicate median and 25/75 percentiles and whiskers indicate 10/90 percentiles. P values were determined by Kruskal-Wallis test followed by a Dunn's post hoc test (*<0.05; **<0.01; ***<0.001) or by Mann Whitney U test (# <0.05). Mann Whitney U test was only applied between CD subgroups and results are only shown if the Dunn's post hoc test did not show any significance.

Anti-TNF-α treatment in CD patients was associated with decreased levels of ASCA IgG and IgA as well as anti-*E. coli* IgG, whereas anti-food and anti-*B. fragilis* IgG and IgA levels were not different in anti-TNF-α treated patients (Figure 4). The duration of anti-TNF-α treatment was 13±10 months (mean ± SD, n = 15). The indication was steroid-dependent/-refractory inflammatory activity that could not be sufficiently treated with immunosuppressants (n = 10), fistulas (n = 3), post-surgery prophylaxis (n = 1) and CD-associated granulomatous chelitis (n = 1).

In our analysis the following parameters were not significantly associated with the Ab levels measured in this study: age at disease onset, disease location, history of surgery, clinical disease activity scores (HBS for CD and CAI for UC), serum CRP levels, WBC, ESR, presence of food intolerance, treatment with steroids or immunosuppressants. Consistent with the fact that specific Ab levels are independent of the clinical disease activity, the amount of anti-food and anti-microbial Abs found in serum were relatively stable over time in patients investigated at different time points (Figure S4).

### Anti-food and anti-microbial IgG and IgA in feces

Concentrations of total fecal IgA were about 1000 times higher than concentrations of fecal IgG which were below the detection limit in about 30% of the cases (Figure S5). Concentrations of fecal IgG and IgA were not significantly different between controls and patient groups, although IgA levels tended to be higher in patient groups.

**Figure 5:**
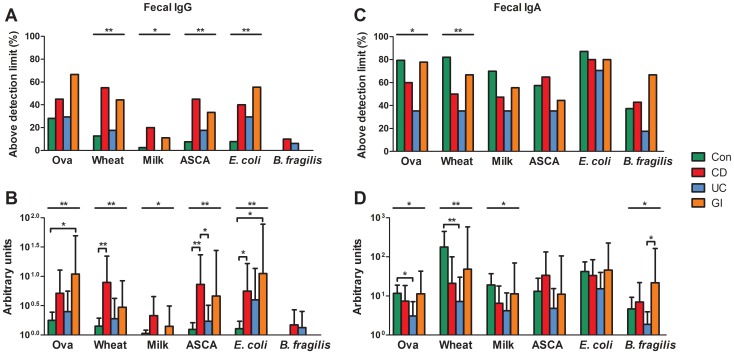
Fecal IgG and IgA levels specific for food and microbial antigens in IBD patients and controls. Specific fecal IgG (A/B) and IgA (C/D) were quantified by ELISA in control patients/healthy controls (Con; n = 39) and patients suffering from CD (n = 20), UC (n = 17) and acute gastroenteritis/colitis (AGE; n = 9). (A/C) Percentage of measurements above the detection limit and (B/D) geometric means ± 95% confidential interval are shown. (A/C) P values (*<0.05; **<0.01) were determined by Chi-square test. (B/D) P values (*<0.05; **<0.01; ***<0.001) were determined by Kruskal-Wallis test followed by a Dunn's post hoc test. The results of the Kruskal-Wallis test are indicated above the graph and the results of the Dunn's post hoc test are indicated directly above the bars.

Specific fecal IgG were only detectable in 0–28% of controls, whereas the percentage of samples above the detection limit and the geometric means of absolute values were generally higher in CD and AGE patients for all tested antigens (Figure 5A/B). UC patients had similar amounts of fecal specific IgG levels compared to controls with only a slight but not significant increase in anti-*E. coli* IgG levels. The highest levels of specific anti-food and anti-microbial IgA were found in controls and AGE patients (Figure 5 C/D). CD patients had lower levels of specific IgA for all tested antigens except for ASCA although the differences did not reach statistical significance in the post-hoc test. The lowest IgA levels for all antigens were found in UC patients. The overall results were statistically different in the Kruskal-Wallis analysis for the three anti-food and anti-*B. fragilis* IgA, whereas the results for ovalbumin and wheat were only significantly different between controls and UC patients in the post-hoc analysis.

There was no statistical significant correlation between serum and fecal Ab levels in controls and UC patients. In CD patients we found a positive correlation between serum and fecal Ab levels for all anti-food IgGs and IgAs, except for anti-ovalbumin IgA (Figure S6).

## Discussion

Immune tolerance towards luminal antigens is a key regulatory mechanism to maintain the homeostasis at the intestinal mucosal surface. Innate and adaptive immune responses towards commensals have been suggested to play a role in triggering chronic inflammation in IBD [24], which is associated with a breakdown of immune tolerance. A number of IBD patients suffer from food intolerances showing an improvement of well-being by avoiding special nutritive components [25]–[27]. However, the relevance of the relation between an exaggerated immune response against food antigens and the occurrence of food intolerance in IBD patients remains an open question.

In the present study we analysed in parallel serum and fecal Abs specific for dietary and microbial antigens in a cohort of IBD patients and controls (for summary of the results see table 1). The objective of this study was to investigate whether increased local or systemic levels of anti-microbial Abs in IBD patients correlate with levels of anti-food Abs, which would argue for a general loss of immune tolerance towards luminal antigens. However, our results reveal that on the systemic level only Abs directed against some microbial antigens are elevated in CD but not UC patients, whereas anti-food Abs showed no general alterations in IBD patients. Anti-food IgA levels were slightly elevated in CD patients with a stricturing/penetrating phenotype. These patients have also elevated anti-microbial Abs levels, a finding that confirms the results of several former studies [6], [10], [28], [29]. Altogether, these findings argue for a more general loss of tolerance to intestinal antigens in severely affected CD patients that very likely have a stronger disturbance of the intestinal barrier than CD patients without stricturing and penetrating lesions. Those patients generally have a milder disease course and/or shorter disease duration and only show enhanced Ab levels towards selected microbial antigens.

**Table 1:** Summary of results. Ab levels compared to controls.

	Crohn's disease	Ulcerative colitis
Serum anti-food IgG/IgA	↔ 1 ^,^ 2	↔ 2
Serum anti-microbial IgG/IgA	↑ 3 ^,^ 4	↔
Fecal anti-food/-microbial IgG	↑	↔
Fecal anti-food/-microbial IgA	↔	↓

1 Slightly increased anti-food IgA levels in CD patients with a structuring/penetrating phenotype.

2 Decreased anti-food IgG levels in CD and UC patients with artopathy.

3 Only some microbial antigens.

4 Patients receiving anti-TNFα treatment have lower Ab levels.

Surprisingly, anti-food IgG levels were decreased in CD and UC patients with arthropathy, an extraintestinal manifestation of IBD which occurs in about 30% of patients. The reason for these findings remains completely elusive and requires further investigation. One recent study demonstrates higher anti-food Ab levels in jejunal fluids of patients with rheumatoid arthritis [30], however we are not aware of further studies analysing antibodies directed against food antigens in patients with arthritis or arthropathy.

Biologicals targeting TNFα are currently the most effective treatment for IBD. We found lower levels of anti-microbial Abs in CD patients receiving anti-TNFα treatment compared to CD patients that did not receive anti-TNFα treatment. The Ab levels in patients on anti-TNFα treatment were almost in the range of healthy controls. We may interpret this finding with caution because our results do not include longitudinal data from patients before and after introduction of anti-TNFα treatment and because our cohort of anti-TNFα CD patients is relatively small and includes only 15 cases. Furthermore, a recent study that included longitudinal sampling did not report a decrease in ASCA levels in CD patients after introducing anti-TNFα [31]. However, in the latter study only in 12 out of 45 CD patients ASCA antibodies could be detected. If anti-TNFa treatment affect anti-microbial Ab levels, it is unlikely to be solely explained by the effects of this treatment on dampening active disease and acute inflammation since Ab levels were independent of clinical disease activity scores and inflammatory markers. However, anti-TNFα therapy is most effective in the induction of long term remission and mucosal healing. It is clearly conceivable that a long-term suppression of intestinal inflammation and repair of intestinal barrier defects may lead to decreased anti-microbial adaptive immune responses including ASCA levels. In line with this hypothesis it is important to note that Gluten-free diet has been shown to reduce ASCA levels in patients with celiac disease in which ASCA levels are also slightly increased [32]. Other mechansims such as direct targeting of memory T and B lymphocytes by anti-TNFα Abs may also play a role in reduction of ASCA levels. It would be interesting to confirm our results in larger cohorts including longitudinal samples and to further decipher the underlying mechanisms.

Fecal and serum Ab levels did not correlate in controls and UC patients, whereas higher correlations were detected in CD patients in particular for anti-food Abs. On the one hand, fecal Ab levels showed specific patterns in different patient groups with enhanced specific IgG against all antigens in patients with CD and acute gastroenteritis or colitis. On the other hand, specific IgA levels against all antigens were significantly decreased in UC patients. Fecal Ab levels were frequently not detectable or only just above the detection limit. There might be several reasons for the low specific Ab levels in fecal samples including the fact that the majority of fecal IgGs may be degraded or bound to commensals as it has been reported recently [33]. However, our results are consistent with previous reports showing similar patterns for different microbial Ab levels in mucosal washings obtained during endoscopy [11], [34]. It is unclear whether increased specific fecal IgG levels mainly seen in patients with CD and AGE result from higher local production or serum leakage. However, higher local production of anti-microbial Abs has been strongly suggested by the study of Macpherson et al., since they did not find elevated fecal IgG levels directed against bacterial strains exclusively found on the skin flora [11]. It is unlikely that decreased specific IgA levels in UC patients are caused by higher fluid contents in the stool, since total IgA levels are slightly elevated and we did not find any correlation between specific fecal IgA and disease activity. Furthermore, the fact that total fecal IgG and IgA levels did not significantly differ among controls and patient groups whereas we found significant differences in Abs specific for luminal antigens further argues for disease-specific patterns of Ab-production rather than non-specific common mechanisms for up- and down-regulation of IgGs and IgAs. The underlying mechanism and functional consequence of our results are unclear. However, it is interesting to note that CD is associated with increased specific IgGs, which are supposed to have proinflammatory functions and are primed exclusively by T cell-dependent mechanisms, whereas UC is associated with decreased specific IgA production, which may have anti-inflammatory functions due to immune exclusion.

Overall, the profile of systemic and local Abs specific for microbial and food antigens reveals a complex picture. Different patient groups and controls showed a distinct Ab pattern, which argues for the fact that adaptive immune responses towards luminal antigens play a distinct role in these diseases. However, the results show that the simple assumption that gut inflammation and barrier defects cause higher IgG and IgA production against luminal antigens is wrong. The results of this and other studies raise several questions. For example, it remains not fully understood why Ab production towards distinct microbial and food antigens is differentially regulated, and why CD patients have elevated serum reactivity towards multiple microbial antigens [6], [29], whereas this phenomenon seems to be less obvious for UC patients, although the microbial load is much higher in the colon [8]. We may speculate that the association of microbial antigens with immune-stimulating molecules such as lipopolysaccharides and peptidoglycans have a major influence on their potential to induce adaptive immune responses. Furthermore, lymphoid aggregates are more frequently found in the small intestine than in the colon. This fact might be one reason for increased Ab production against luminal antigens in CD patients that often show small intestinal and transmural involvement, whereas this is not the case for colonic inflammation restricted to the mucosa as found in UC patients.

One important finding of our study is that we did not find any correlation between food-specific Ab levels and clinical symptoms. Our data does not support the idea that measurements of anti-food specific IgG or IgA is of any clinical relevance for patients suffering from CD or UC. In particular, it does not predict the presence of food intolerance. It is also unclear whether anti-microbial Abs have any disease-aggravating effect or whether they are only a concomitant phenomenon of chronic gut inflammation. Since we found detectable Ab levels in many individuals it is even questionable whether high Ab levels are associated with a loss of tolerance. Furthermore, some of the measured Abs might be cross-reacting Abs that were primarily formed against other antigens. For example it has been shown that ASCA do cross-react with Candida antigens [35] and that ASCA levels do not correlate with the presence of *S. cervisiae* in the intestinal tract of patients [36]. Acute exacerbation of disease does not correlate with antibody levels, however enhanced ASCA and other anti-microbial Ab levels correlate with more complicated disease courses and anti-TNF-α therapy, It has been shown that microbial-specific IgA mediates gut homeostasis in animal models [37] and might therefore have anti-inflammatory functions in IBD. However, it has been reported microbial-antigen specific T cells have been shown to induce chronic gut inflammation in experimental models [38], [39], which provides some evidence that a loss of adaptive immune tolerance towards microbial antigens can exaggerate chronic intestinal inflammation.

In summary, our study reveals that CD, UC and AGE patients as well as non-inflammatory controls have distinct patterns of IgG and IgA against food and microbial antigens in serum and feces suggesting differentially regulated immune responses towards intestinal antigens. However, food-specific Abs have not yet been proven to be a valuable biomarker for IBD or food intolerance.

## Supporting Information

Figure S1 **Correlation of specific serum IgG and IgA levels.** Correlation of serum IgG (A) and serum IgA (B) levels specific for different food and microbial antigens in single individuals. Correlation of serum IgG and IgA levels against the same antigen (C). Y axis indicates Spearmen's correlation coefficient r_s_. Correlations are shown separately for control patients/healthy controls (Con; n = 61) and patients suffering from CD (n = 52) and UC (n = 29). Ova, ovalbumin; Mi, Milk; Wh, wheat; ASCA, anti-*Saccharomyces cerevisiae* antibodies; EC, *Escherichia coli*; Bac, *Bacteroides fragilis*. Significances are indicated above bars (*<0.05; **<0.01; ***<0.001).
(TIF)

Figure S2 **Correlation of age and specific serum IgG and IgA levels.** Correlation of serum IgG and serum IgA levels specific for different food and microbial antigens and age of patients and controls. Correlations are shown separately for control patients/healthy controls (Con; n = 61) and patients suffering from CD (n = 52) and UC (n = 29). Significances are indicated above bars (*<0.05; **<0.01; ***<0.001).
(TIF)

Figure S3 **Anti-food and anti-microbial serum IgG and IgA levels in UC patients with or without arthropathy and controls.** Specific serum IgG (A) and IgA (B) were quantified by ELISA in control patients/healthy controls (n = 61) and UC patients without (n = 20) and with (n = 9) current arthropathy. Boxes indicating median and 25/75 percentiles and whiskers indicating 10/90 percentiles are shown. Kruskal-Wallis test did not show any significant difference between the three groups. Mann Whitney U test was applied between UC subgroups (#<0.05).
(TIF)

Figure S4 **Specific serum IgG and IgA levels in IBD patients at different time points.** Anti-food and anti-microbial serum IgG (A) and IgA (B) were measured in 11 CD and 6 UC patients at two different visits (91±44 days between the two visits, mean ± SD). r_s_ indicates Spearman's correlation coefficient between the two measurements.
(TIF)

Figure S5 **Total fecal IgG and IgA levels in IBD patients and controls.** Total IgG (A) and IgA (B) in fecal homogenates were quantified by ELISA in control patients/healthy controls (Con; n = 39) and patients suffering from CD (n = 20), UC (n = 17) or acute gastroenteritis/colitis (AGE; n = 9). Geometric means ± 95% confidential intervals are shown.
(TIF)

Figure S6 **Correlation of serum vs. fecal antigen-specific IgG and IgA levels.** Correlations are shown separately for control patients/healthy controls (Con; n = 39) and patients suffering from CD (n = 20) and UC (n = 17). Significances are indicated above bars (*<0.05; **<0.01).
(TIF)

Table S1 **Patient characteristics.**
(DOCX)

Table S2 **Detailed characteristics of IBD patients.**
(DOCX)
