# Supplementary figures and images for: Distinct Patterns of IgG and IgA against Food and Microbial Antigens in Serum and Feces of Patients with Inflammatory Bowel Diseases
Source: PLoS One. 2014 Sep 12;9(9):e106750. doi: 10.1371/journal.pone.0106750 (PMC4162554; doi:10.1371/journal.pone.0106750)

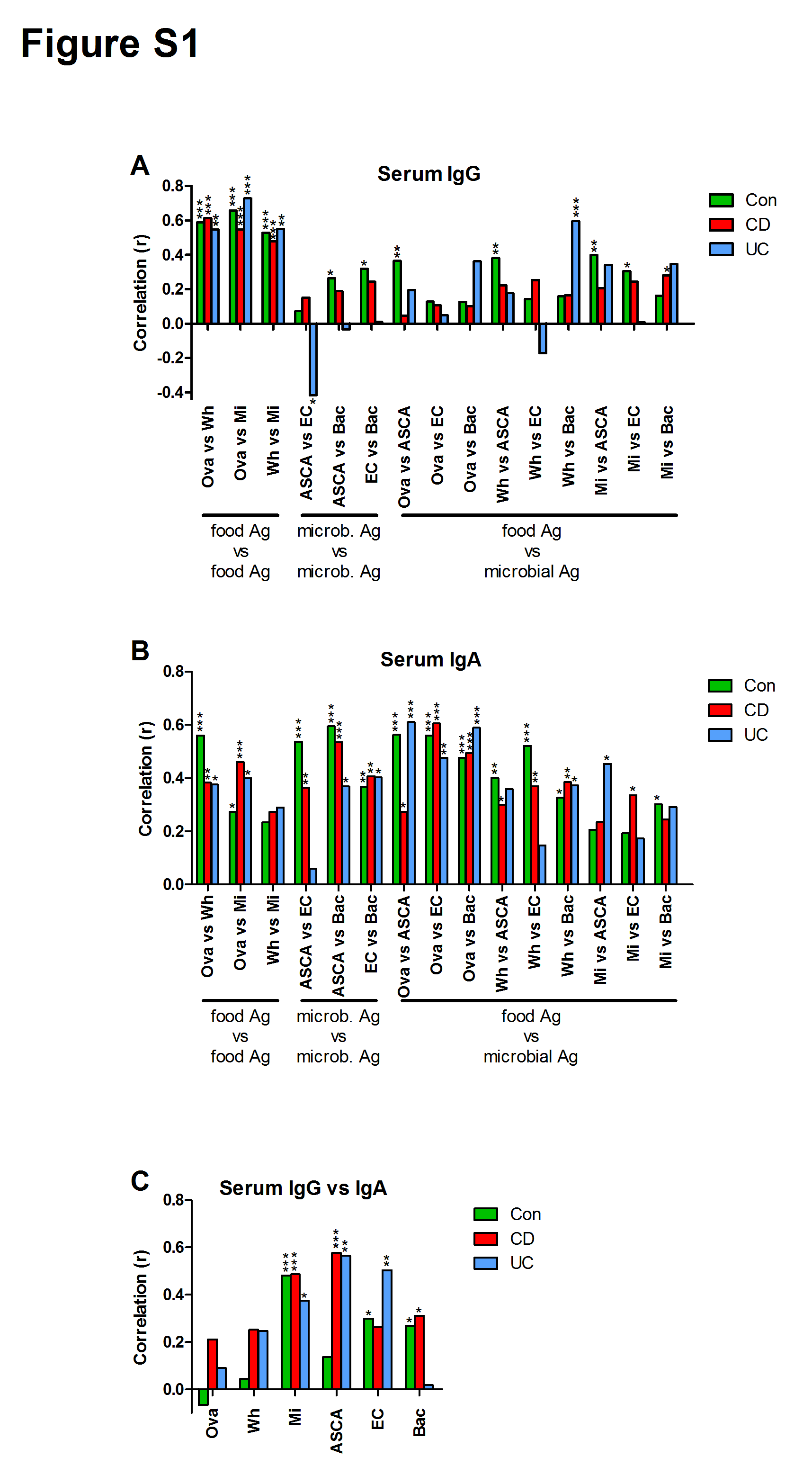

Supplement: Figure S1 — Correlation of specific serum IgG and IgA levels. Correlation of serum IgG (A) and serum IgA (B) levels specific for different food and microbial antigens in single individuals. Correlation of serum IgG and IgA levels against the same antigen (C). Y axis indicates Spearmen's correlation coefficient rs. Correlations are shown separately for control patients/healthy controls (Con; n = 61) and patients suffering from CD (n = 52) and UC (n = 29). Ova, ovalbumin; Mi, Milk; Wh, wheat; ASCA, anti-Saccharomyces cerevisiae antibodies; EC, Escherichia coli; Bac, Bacteroides fragilis. Significances are indicated above bars (*<0.05; **<0.01; ***<0.001). (TIF) [file pone.0106750.s001.tif]

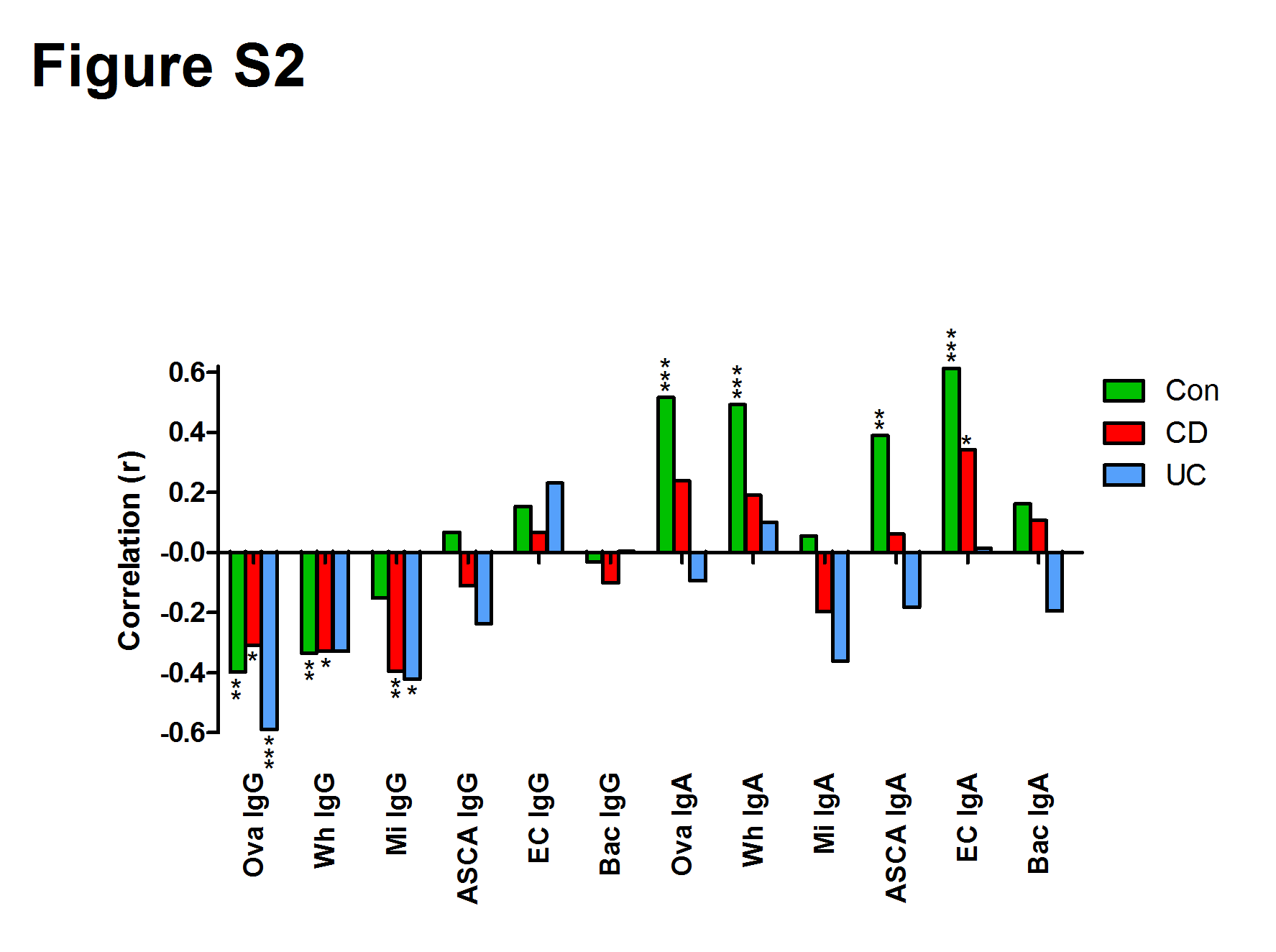

Supplement: Figure S2 — Correlation of age and specific serum IgG and IgA levels. Correlation of serum IgG and serum IgA levels specific for different food and microbial antigens and age of patients and controls. Correlations are shown separately for control patients/healthy controls (Con; n = 61) and patients suffering from CD (n = 52) and UC (n = 29). Significances are indicated above bars (*<0.05; **<0.01; ***<0.001). (TIF) [file pone.0106750.s002.tif]

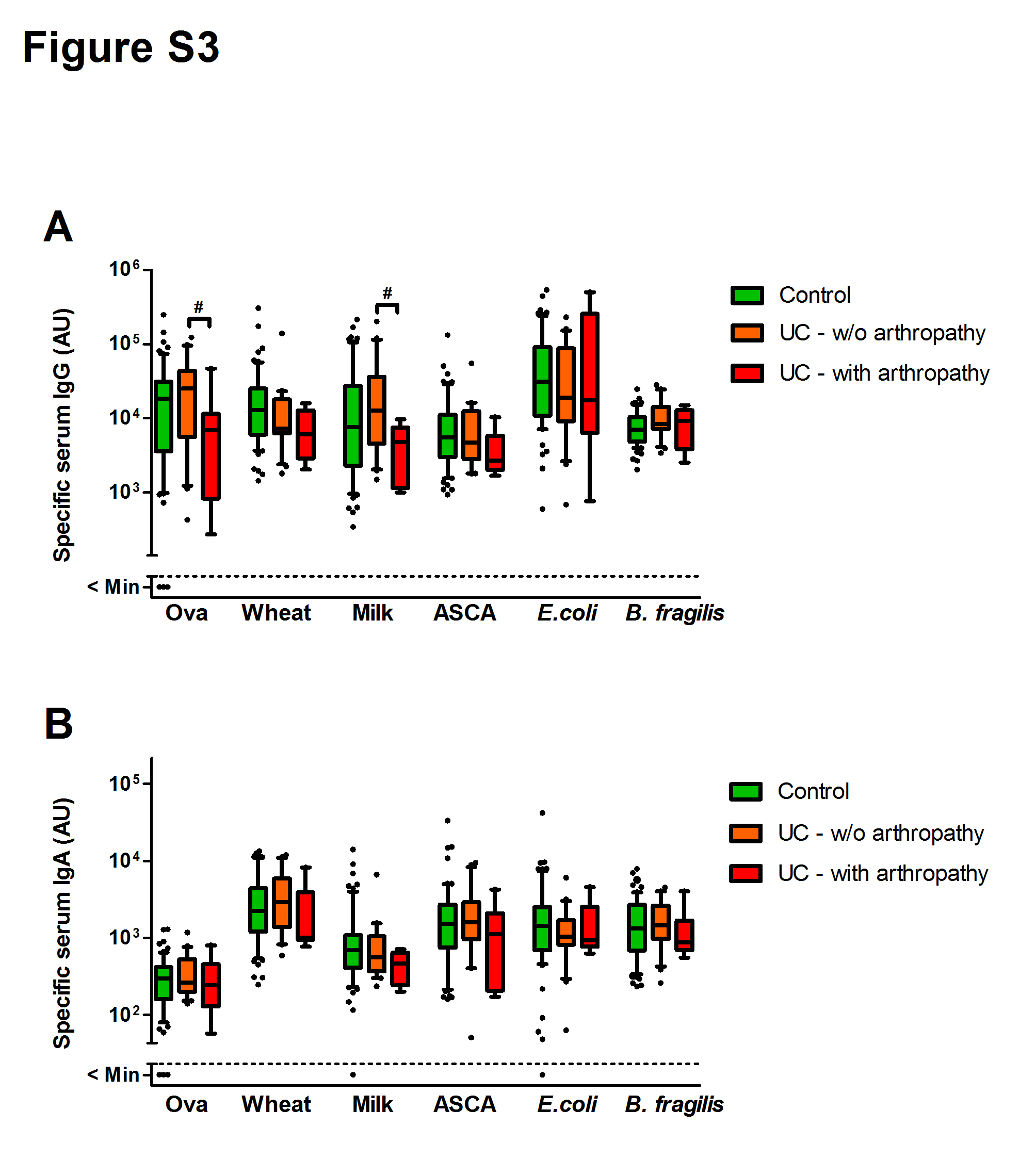

Supplement: Figure S3 — Anti-food and anti-microbial serum IgG and IgA levels in UC patients with or without arthropathy and controls. Specific serum IgG (A) and IgA (B) were quantified by ELISA in control patients/healthy controls (n = 61) and UC patients without (n = 20) and with (n = 9) current arthropathy. Boxes indicating median and 25/75 percentiles and whiskers indicating 10/90 percentiles are shown. Kruskal-Wallis test did not show any significant difference between the three groups. Mann Whitney U test was applied between UC subgroups (#<0.05). (TIF) [file pone.0106750.s003.tif]

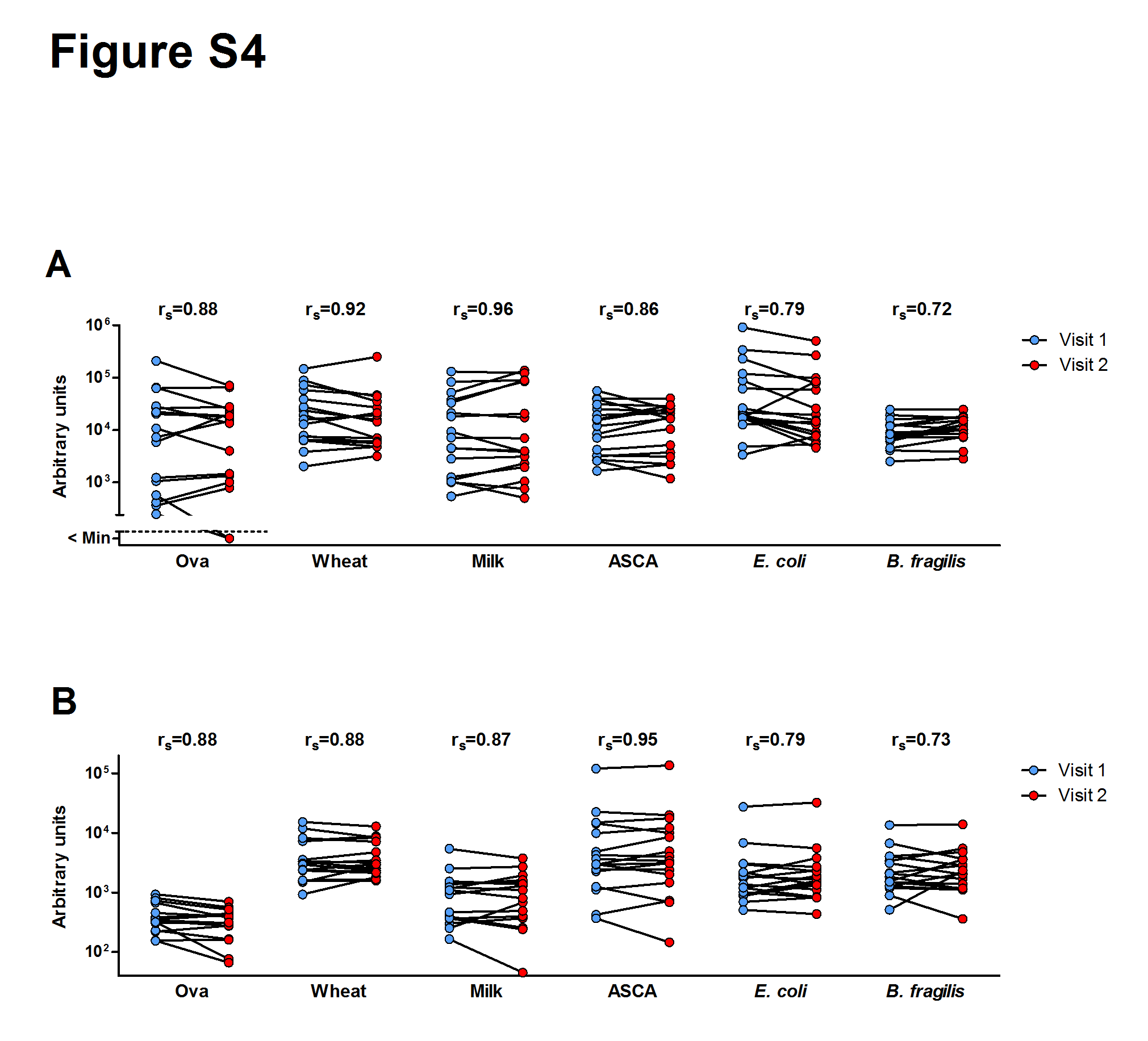

Supplement: Figure S4 — Specific serum IgG and IgA levels in IBD patients at different time points. Anti-food and anti-microbial serum IgG (A) and IgA (B) were measured in 11 CD and 6 UC patients at two different visits (91±44 days between the two visits, mean ± SD). rs indicates Spearman's correlation coefficient between the two measurements. (TIF) [file pone.0106750.s004.tif]

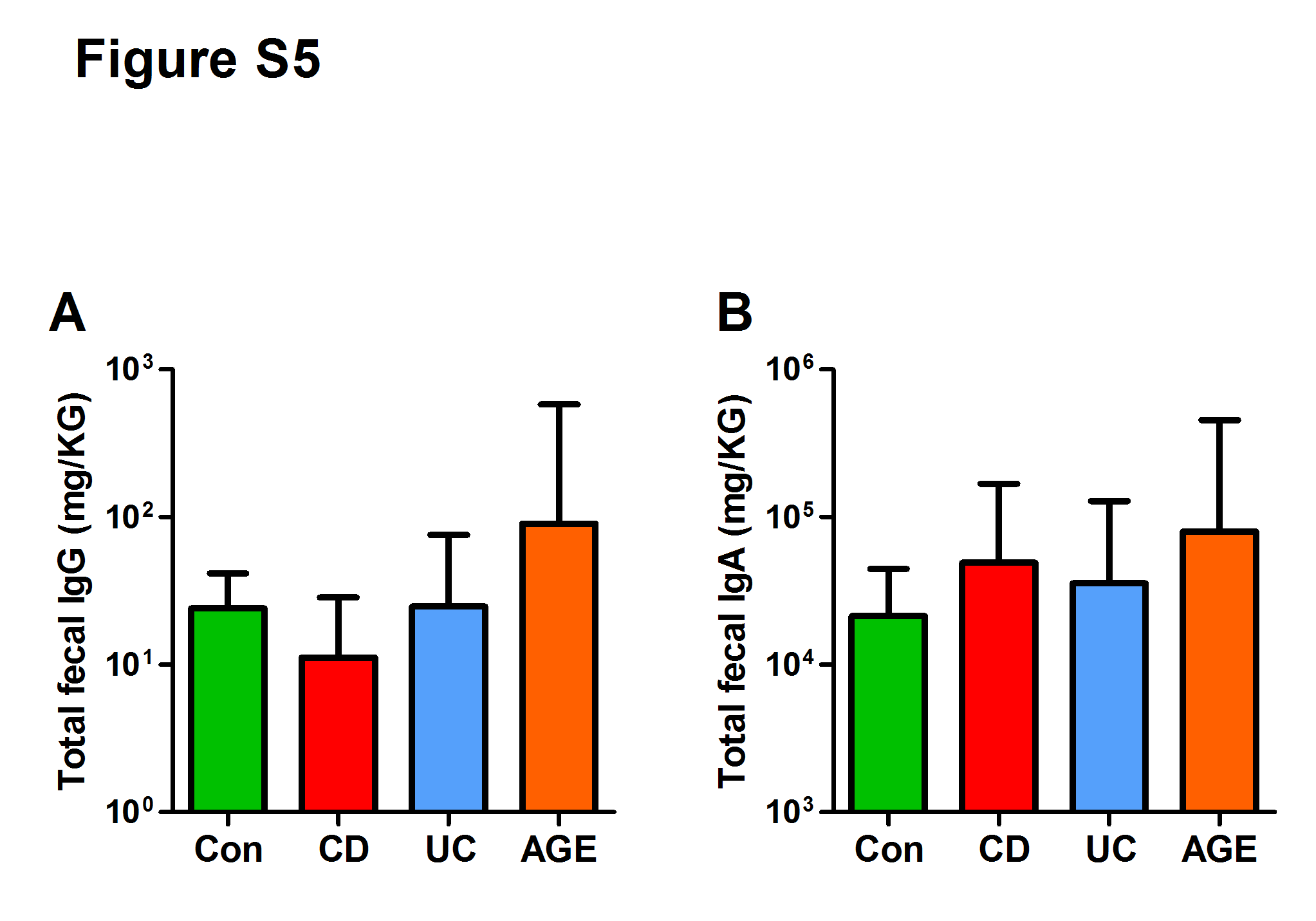

Supplement: Figure S5 — Total fecal IgG and IgA levels in IBD patients and controls. Total IgG (A) and IgA (B) in fecal homogenates were quantified by ELISA in control patients/healthy controls (Con; n = 39) and patients suffering from CD (n = 20), UC (n = 17) or acute gastroenteritis/colitis (AGE; n = 9). Geometric means ± 95% confidential intervals are shown. (TIF) [file pone.0106750.s005.tif]

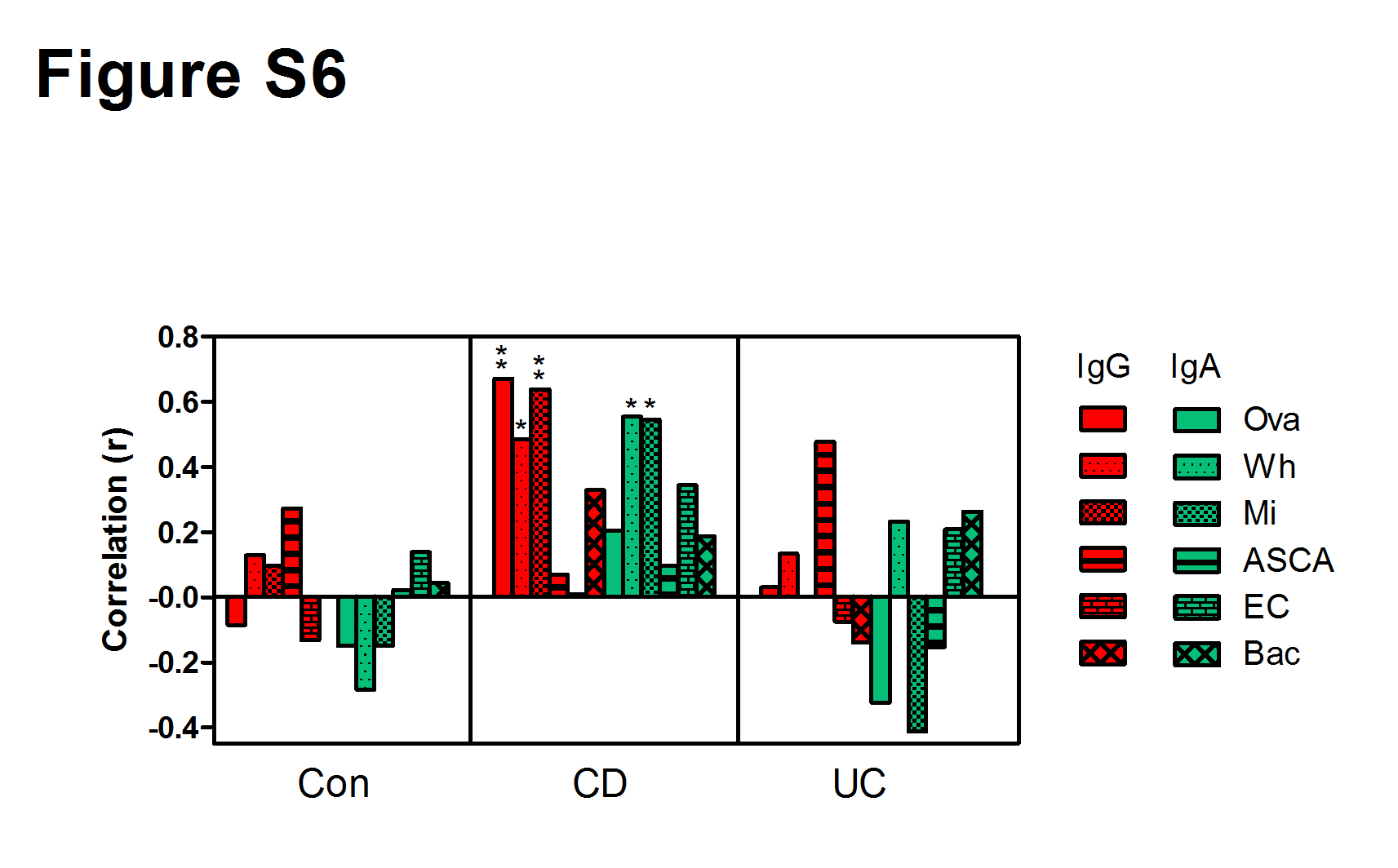

Supplement: Figure S6 — Correlation of serum vs. fecal antigen-specific IgG and IgA levels. Correlations are shown separately for control patients/healthy controls (Con; n = 39) and patients suffering from CD (n = 20) and UC (n = 17). Significances are indicated above bars (*<0.05; **<0.01). (TIF) [file pone.0106750.s006.tif]
