# Supplementary material for: Distinct Patterns of IgG and IgA against Food and Microbial Antigens in Serum and Feces of Patients with Inflammatory Bowel Diseases
Source: PLoS One. 2014 Sep 12;9(9):e106750. doi: 10.1371/journal.pone.0106750 (PMC4162554; doi:10.1371/journal.pone.0106750)
Supplement: Table S1 — Patient characteristics. (DOCX) [file pone.0106750.s007.docx]

**Table S1.** Patient characteristics

|  | n | Age - median (interquartile range) | Female sex - no. (%) |
| --- | --- | --- | --- |
| **Controls (Con) [a + b]** | **61** | **37 (25 - 56)** | **34 (56)** |
| 1. Control patients ^1^ | 35 | 55 (43 - 67) | 18 (51) |
| 1. Healthy volunteers | 26 | 25 (23 - 27) | 16 (62) |
| **Crohn’s disease (CD)** | **52** | **33 (25 - 46)** | **25 (48)** |
| **Ulcerative Colitis (UC)** | **29** | **35 (27 - 52)** | **11 (38)** |
| **Non-IBD gastrointestinal inflammation (AGE) ^2^** | **12** | **41 (24 - 57)** | **6 (50)** |

1. Main diagnoses: gastrointestinal bleeding or anemia because of iron or folic acid deficiency (n = 9), gastritis and reflux disease (n = 9), tumors and tumor-like lesions (n = 7), liver cirrhosis (n = 4), pancreatic disease (n = 3), irritable bowel syndrome (n = 2), cardiac diseases (n = 2)
2. Diagnoses: acute gastroenteritis (n = 6; no pathogen identified); acute colitis (n = 6; 2 *Clostridium difficile*; 1 cytomegalovirus; 1 *Campylobacter jejuni*; 2 no pathogen identified)
