# Supplementary material for: Distinct Patterns of IgG and IgA against Food and Microbial Antigens in Serum and Feces of Patients with Inflammatory Bowel Diseases
Source: PLoS One. 2014 Sep 12;9(9):e106750. doi: 10.1371/journal.pone.0106750 (PMC4162554; doi:10.1371/journal.pone.0106750)
Supplement: Table S2 — Detailed characteristics of IBD patients. (DOCX) [file pone.0106750.s008.docx]

**Table S2.** Detailed characteristics of IBD patients

|  | **Crohn’s disease** | **Ulcerative colitis** |
| --- | --- | --- |
| Inactive disease | 31 (HBI < 5) | 12 (CAI < 6) |
| Active disease | 20 (HBI ≥ 5) | 17 (CAI ≥ 6) |
| Localization ^1^ | L1 (ileal): 7 | E1 (proctitis): 1 |
|  | L2 (colonic): 7 | E2 (left side): 16 |
|  | L3 (ileocolonic): 36 | E3 (pancolitis): 10 |
|  | *L4 (additional upper disease): 11 | Pouchitis: 2 |
| Behaviour ^1^ | B1 (non-stricturing/penetrating): 17 |  |
|  | B2 (stricturing): 7 |  |
|  | B3 (penetrating): 5 |  |
|  | B2B3 (stricturing+penetrating): 21 |  |
|  | *Perianal disease: 15 |  |
| Age at diagnosis | A1 (≤ 16 years): 7 | A1: 7 |
|  | A2 (17 - 40 years): 38 | A2: 14 |
|  | A3 (> 40 years): 6 | A3: 8 |
| Duration ^2^ | 8.2 (3.0 - 16.1) years | 9.1 (3.3 - 15.7) years |
| Arthropathy ^3^ | 12 (24 %) | 9 (31 %) |
| anti-TNF treatment | 15 (29 %) | 7 (24 %) |

^1^ One CD patient not documented; ^2^ median ± interquartile range; ^3^ current symptoms; * modifier
